# Optimization of Foams—Polypropylene Fiber-Reinforced Concrete Mixtures Dedicated for 3D Printing

**DOI:** 10.3390/ma17164106

**Published:** 2024-08-19

**Authors:** Magdalena Rudziewicz, Marcin Maroszek, Kinga Setlak, Mateusz Góra, Marek Hebda

**Affiliations:** Faculty of Materials Engineering and Physics, Cracow University of Technology, Warszawska 24, 31-155 Kraków, Poland; magdalena.rudziewicz@doktorant.pk.edu.pl (M.R.); marcin.maroszek@doktorant.pk.edu.pl (M.M.); kinga.plawecka@pk.edu.pl (K.S.); mateusz.gora@doktorant.pk.edu.pl (M.G.)

**Keywords:** foamed concrete, additive manufacturing, fiber reinforcement

## Abstract

The continued global urbanization of the world is driving the development of the construction industry. In order to protect the environment, intensive research has been carried out in recent years on the development of sustainable materials and ecological construction methods. Scientific research often focuses on developing building materials that are renewable, energy-efficient, and have minimal impact on the environment throughout their life cycle. Therefore, this article presents research results aimed at developing a concrete mixture using cement with reduced CO_2_ emissions. In the context of increasing ecological awareness and in line with European Union policy, the development of a mixture based on environmentally friendly cement is of key importance for the future development of the construction industry. The article compares the physical properties of two mixtures, their foaming possibilities, and the influence of the added polypropylene (PP) fibers on the strength properties of the produced composites. It was found that bending strength and compressive strength were highest in the material with silica fume and aluminum powder at 5.36 MPa and 28.76 MPa, respectively. Microscopic analysis revealed significant pore structure differences, with aluminum foamed samples having regular pores and hydrogen peroxide foamed samples having irregular pores. Optimizing aluminum powder and water content improved the materials’ strength, crucial for maintaining usability and achieving effective 3D printing. The obtained results are important in the development of research focused on the optimization of 3D printing technology using concrete.

## 1. Introduction

### 1.1. Aerated Concrete

Foamed concrete is gaining prominence as an increasingly favored alternative to conventional concrete in residential construction applications. This material is utilized for its acoustic and thermal insulating properties, alongside its notably high fire resistance. Furthermore, aerated concrete demonstrates potential in reducing the weight of prefabricated building elements and the mitigation of seismic impact on structural integrity. The utilization of prefabricated elements fabricated from foamed concrete presents a cost-effective solution compared to those constructed from traditional concrete, thereby contributing to overall construction cost reduction. Attributable to its advantageous properties, foamed concrete stands as a promising candidate for application in 3D concrete printing endeavors. In contrast to conventional lightweight concrete with densities ranging from 1000 to 2000 kg/m^3^ and compressive strengths between 1 to 100 MPa [1,2] foamed concrete exhibits notably lower densities, potentially reaching up to 400 kg/m^3^ [3]. Elements fabricated from foamed materials are distinguished by notably lower density compared to conventional solid counterparts. Moreover, it is possible to use various raw materials and foaming methods [4,5]. One manufacturing approach involves incorporating pre-prepared foam into cement paste to introduce porosity within the composite. Alternatively, aerated concrete can be generated by incorporating chemical foaming agents into the concrete matrix [6]. During the reaction between aluminum powder (as the foaming agent) and the calcium hydroxide generated during cement hydration, hydrogen gas bubbles are produced [7]. Achieving a stable aerated concrete (utilizing aluminum powder or hydrogen peroxide as the foaming agent) amenable to pre-processing, pumping, and 3D printing poses a formidable challenge, with scant research hitherto addressing this exigency. Alternatively, traditional concrete formulation has undergone extensive scrutiny regarding the impact of the aluminum (Al) content on the compressive strength of aerated concrete [8,9,10,11,12]. Shabbar et al. demonstrated a decrease in the compressive strength of aerated concrete (AC) specimens with escalating aluminum powder content across samples containing 0.25%, 0.5%, 0.75%, and 1% Al. The internal surfaces of specimens featuring aluminum (Al) powder concentrations ranging from 0.5 to 1% exhibited irregularly shaped pores of greater dimensions than those apparent in specimens with 0.25% content [2]. Guglielmi et al. observed an initial marginal decline in compressive strength followed by augmentation as porosity diminished. Moreover, it was discerned that excessive aluminum powder hindered pore formation due to interactions with hydrogen peroxide [13]. Additionally, aerated concrete, characterized by a density of 686 kg/m^3^ and a compressive strength of approximately 6.3 MPa, was successfully produced [14].

In the realm of advanced building materials, aerated concrete finds extensive application in external building insulation, internal gap filling, and fire protection systems. A judicious selection of the foaming agent and cement matrix profoundly impacts the properties of foam concrete. Hydrogen peroxide (H_2_O_2_) emerges as a particularly advantageous blowing agent owing to its controlled decomposition kinetics, rendering it a cost-effective and environmentally friendly alternative to aluminum powder and conventional blowing agents. Hydrogen peroxide, an inorganic liquid, exhibits complete miscibility with water in any proportion. Its decomposition rate can be effectively regulated using catalysts, such as manganese dioxide and permanganate. As a fully ecological product, its decomposition yields water and oxygen as by-products. Several researchers, including Pan et al. [15], Rooyen et al. [3], and Wu et al. [16], have demonstrated the use of H_2_O_2_ as a foaming agent for conventional concrete based on Portland cement, resulting in concrete foam with densities ranging from 150 kg/m^3^ to 300 kg/m^3^ [15]. However, it is essential to consider potential challenges when utilizing foamed concrete in additive manufacturing technology. Foamed concrete is prone to deformation in the lower layers of prints due to the weight exerted by subsequent layers [17,18]. Therefore, the initial steps in designing a foamed material tailored for 3D printing and developing production methods should involve verifying the stability and consistency of the prepared foamed materials and assessing their mechanical properties. The development of foam materials in concrete technology has been limited due to their brittleness and excessive susceptibility to cracks. One method to limit crack propagation in concrete is the incorporation of fibers, which serve as bridging mechanisms and enhance the material’s resistance to cracking.

### 1.2. Fibers

In order to enhance the viscosity and consistency of the material for 3D concrete printing (3DCP), fibers can be introduced as additives. Their incorporation aims to improve properties related to maintaining layer shape after extrusion and to limit deformation resulting from the deposition of subsequent layers [19]. The addition of fibers plays a crucial role in preserving the geometry of the concrete throughout the printing process, thereby increasing the accuracy and precision of the resulting prints [20]. In traditional concrete structures, fibers are commonly used to improve the performance of manufactured elements. The most frequently used types of fibers include small synthetic fibers (up to 75 mm in length) known for their resistance to various factors, including oxidation. Carbon [21], basalt [22], glass [23], polypropylene [13,24], steel [25], and polyvinyl alcohol (PVA) [26] fibers are among the popular options. Fibers can be categorized into microfibers (with a diameter of less than 0.32 mm and a length of less than 30 mm) and macrofibers (with a diameter of more than 0.32 mm and a length of more than 30 mm) [27]. The introduction of fibers can modify the flow characteristics of concrete, thereby directly influencing its pumpability during the 3D printing process. Research conducted thus far indicates that the addition of nanosilica (NS) and polypropylene (PP) fibers to gypsum formulations can significantly enhance printability and mitigate the observed anisotropy in printed samples. This improvement is attributed to the minimal quantity of NS required [28]. The incorporation of dispersed fibers into concrete also serves as an effective reinforcement method, particularly in experiments involving additive manufacturing technology [29,30,31]. For instance, studies on 3D printing with geopolymers reinforced with polypropylene (PP) fibers demonstrated enhanced resistance to deformation under a load [32]. Additionally, experiments with fiber-reinforced foamed concrete revealed that adding 0.4% PP fibers significantly improved the compressive strength of the material, increasing it by 35% [33]. Further research indicated that PP fibers also enhanced the bending strength of foamed concrete by 40% [34]. Consequently, the enhanced shape stability and construction possibilities render printed concrete a viable option for implementation in civil infrastructure construction.

The analysis of using fiber-reinforced foamed concrete, specifically with polypropylene fibers, in the context of 3D concrete printing is an innovative approach that could bring significant benefits to the construction industry. Before commencing research in this area, it was essential to thoroughly understand the behavior and consistency of the produced mix. Polypropylene fibers added to concrete can enhance its tensile strength and reduce the risk of cracking. A critical aspect of this research is determining the open time for specific mixes, as this is crucial for the 3D printing process. The open time defines the period during which the mix retains sufficient plasticity for shaping. Additionally, it was important to investigate how the material behaves during flow tests at different intervals after preparation. Flow tests allow the assessment of the mix’s ability to maintain proper consistency and homogeneity during application. Polypropylene fibers can also improve the structural stability of the concrete during the printing process. The research aims to determine the optimal mix proportions that will provide the best mechanical and rheological properties. The results of these studies could lead to the development of new standards and guidelines for 3D concrete printing. The innovation of this approach lies in the synergy between advanced building materials and modern manufacturing technologies. This study provides a preliminary characterization of the constituents and methodologies employed in the preparation of foamed materials designed for 3D concrete printing (3DCP). A comparative analysis was performed to assess the stability and rheological consistency of foamed mixtures in comparison to those incorporating polypropylene fibers, forming the basis for the development of a foamed mixture with dispersed reinforcement optimized for 3DCP. The judicious selection of binders and additives, combined with a comprehensive understanding of the material’s behavior, is essential for establishing the new trajectory required to advance innovations in 3DCP technology.

## 2. Materials and Methods

### 2.1. Tested Materials

The research was carried out using two base materials. The first of them was Binder 1 (B1), which, in addition to Portland cement, contains silica fly dust up to 50%. Binder 2 (B2) contained only standard Portland cement. Foaming agents, such as aluminum powder and hydrogen peroxide H_2_O_2_ at a concentration of 30%, were used to produce foamed concrete. PP-12 polypropylene fiber with a length of 12 mm was also used in the study. The physical properties of the ingredients used in accordance with the data sheets issued by the manufacturers are presented in Table 1, Table 2 and Table 3.

The above-mentioned materials were used to prepare 20 mixtures, which were poured into molds measuring 40 × 40 × 160 mm and 100 × 100 × 100 mm. After demolding, the samples were inspected and found to be free of any visible cracks, pores, or defects. The reference samples were made only from a mixture of base binder (30% for B1 and 25% for B2), river sand (70% for B1 and 65% for B2), and water. The precise values for these parameters are provided in Table 4. To evaluate the impact of foaming agents on materials B1 and B2, both aluminum powder (Al) and hydrogen peroxide (H₂O₂) were analyzed. Additionally, samples of foamed concrete containing 0.4% polypropylene (PP) fibers were produced. The composition and proportions of the prepared samples are detailed in Table 4.

### 2.2. Methods

#### 2.2.1. Slump Flow Test

**Figure 1:**
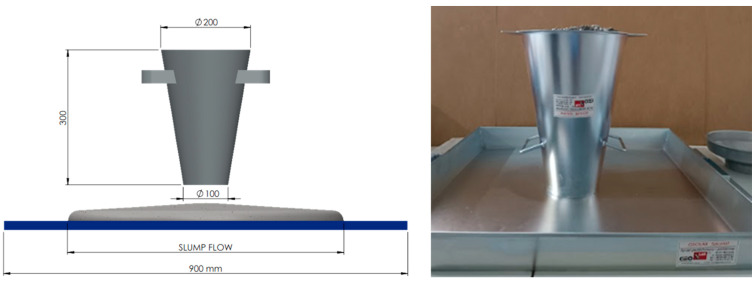
Consistency testing using the falling cone method—free flow. The measurement diagram is one the left, and a photo of the measuring set is on the right.

Consistency tests were conducted using the falling cone method according to the PN-EN 12350-4:2019-08 standard [38]. The method employed involves measuring the flow diameter of the concrete mixture under its own weight after the removal of the mold. The concrete mixture is initially placed in a mold (an Abrams cone) on a flat surface in a single layer, without additional compaction (see Figure 1). Following the removal of the mold, the flow diameter is measured in two perpendicular directions, and the result is reported as the average of these two measurements. This procedure is straightforward to execute both at construction sites and during 3D concrete printing operations. The results obtained from this method enable an assessment of the concrete’s consistency when incorporating either a solid or liquid foaming agent. Moreover, it allows for a quick assessment of the potential benefits of introducing polypropylene fibers into the mixture to improve the rheology and stability of the printing process.

#### 2.2.2. Compressive Strength Tests

**Figure 2:**
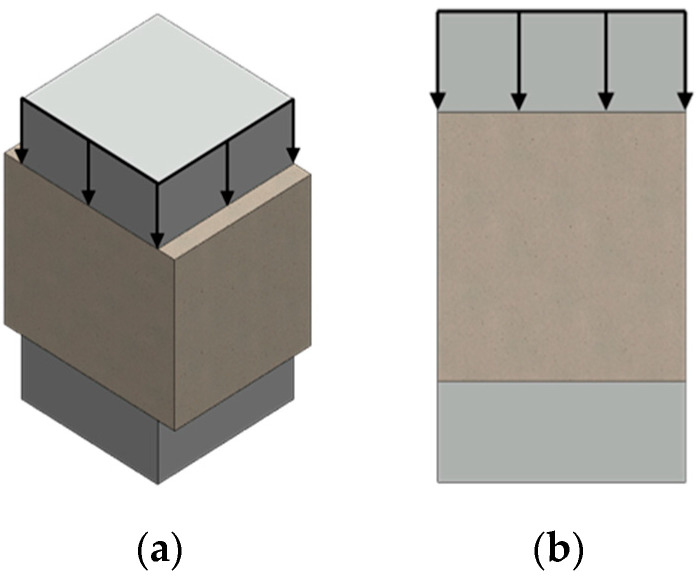
Scheme showing the compressive strength test: (**a**) three-dimensional image; (**b**) cross-section.

Compressive strength tests were carried out in accordance with the PN-EN 12390-3:2019-07 standard [39], using a Matest 3000 kN universal testing machine (Matest, Treviolo, Italy). The dimensions of the prepared samples were 40 × 40 × 40 mm, and the linear speed of force increase of 0.5 MPa/sec increased until the sample was destroyed (Figure 2). The result was estimated from six measurement repetitions for each material variant.

#### 2.2.3. Bending Strength Test

**Figure 3:**
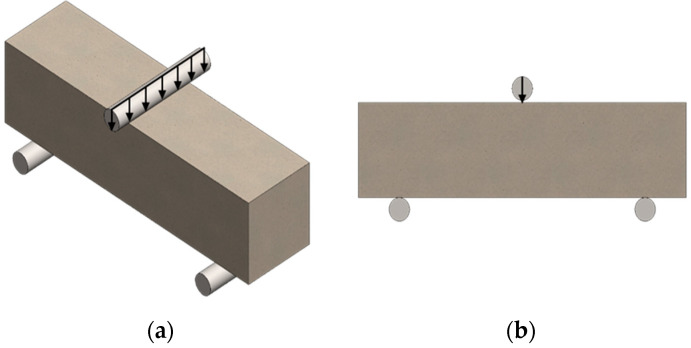
Scheme showing the three-point bending strength test: (**a**) three-dimensional image; (**b**) cross-section.

Flexural strength tests were carried out using a Matest 3000 kN testing machine on 40 × 40 × 160 mm specimens, based on EN 12390-5:2019-08 [40]. The linear speed of the force increase was 0.5 MPa/sec, with distances between supports of 150 mm (Figure 3). The result was estimated from three measurement repetitions for each material variant.

#### 2.2.4. Microscopy Observation

A Techrebal Banito B2920 digital microscope with 100× magnification, integrated with a Techrebal 48 MP camera (Techrebal, Wilczyce, Poland), was used for microscopy observation.

## 3. Results and Discussion

The concrete mix must maintain sufficient plasticity over an extended period, known as the open time, to allow for continuous extrusion without clogging or hardening prematurely. This is essential for the layer-by-layer deposition process. The mix needs to be fluid enough to be extruded through the printer nozzle yet stiff enough to hold its shape once deposited to prevent collapse or deformation of the printed layers. The material must have good buildability, meaning it can support subsequent layers without excessive deformation or collapse. This requires a balance between early strength gain and workable flowability [41]. The mix must be homogeneous and free from segregation or bleeding. For the material to be used in 3D printing, it must be pumpable through the delivery system without causing blockages. This requires the mix to have a low enough viscosity to be pumped but not so low that it affects the buildability. Each printed layer must adhere well to the previous one to ensure structural integrity. This requires the material to retain some level of tackiness or bonding capability during the printing process. The material must exhibit low shrinkage and high resistance to cracking, particularly during the early stages of setting [42,43]. The printed concrete must meet long-term durability requirements, including resistance to environmental factors, such as freeze–thaw cycles, chemical exposure, and moisture ingress. In fiber-reinforced mixes, it is essential to ensure an even distribution of fibers throughout the concrete to avoid weak spots and to enhance mechanical properties, such as tensile and flexural strength [44]. The mix should develop sufficient green strength (initial strength) quickly after extrusion to support the weight of additional layers and to resist deformation under its own weight.

During the 3D printing process using concrete, it is crucial to ensure the stability and durability of individual layers. Therefore, the research focused on verifying the consistency of the base mixture and modifying its composition. The flow measurement of concrete mixtures was carried out 1 min, 20 min, and 40 min after the mixture preparation [45,46,47]. The time interval between the individual tests is due to the fact that, during large-scale production, the interval between layers is significant. Additionally, because of the characteristics of the material, which must be sufficiently cohesive but not too fluid, it is necessary to examine whether printing with the given mixture at these intervals is feasible. It is crucial to verify this information to determine whether it is possible to mix a batch of material for one layer and if it will still have the appropriate consistency to apply a second layer with the same mixture. Similarly, the information obtained from these tests helps confirm whether, after 40 min, the consistency will change enough to allow for the application of another layer. Furthermore, this is particularly important in the context of interlayer forces and their strength [48]. The stability of the foaming process was also assessed depending on the type of foaming agent used. Table 5 shows the results of the slump flow test depending on the composition of the analyzed mixtures. Generally, slightly higher values were measured for samples based on raw material B1 than B2. Concrete mixtures modified with the addition of fibers showed the highest compactness, regardless of the mixing time and type of binder. It was also found that the addition of hydrogen peroxide to the mixture made it impossible to measure its consistency. This effect was independent of the type of raw material used. Comparison of mixtures with similar compositions after identical mixing times reveals clear differences in their properties. Lower flow values were measured each time for mixtures made from B2 raw material. To improve the consistency results of materials made of B1 material, further modifications to the composition of the mixtures are necessary. This result encourages further tests and the process of modifying the composition of the mixture, especially in the context of its suitability in 3D printing processes. In 3DCP technology, efficient mixing and pumping of the prepared mixture is crucial because the material must maintain a balance between being strong enough to maintain the integrity of the layer after printing, yet fluid enough to ensure smooth transport. Concomitantly, it is particularly important to prepare a material with a homogeneous structure; in the event of heterogeneity of the mixture or delamination of the concrete mixture after it dries, gaps and cracks may occur [49]. When segregation occurs and a significant amount of foam remains in the upper part of the layer, insufficient bonding between the layers may result. Cracking may also occur as the material dries and shrinks, often exacerbated by rapid drying [50]. Similarly, structural cracks may appear as a result of excessive loads or damage to the structure. Uneven or excessive settling of material from successive layers can affect long-term exposure to stress, causing permanent deformation. Therefore, achieving the appropriate consistency and stability of the foamed mixture is crucial.

**Figure 4:**
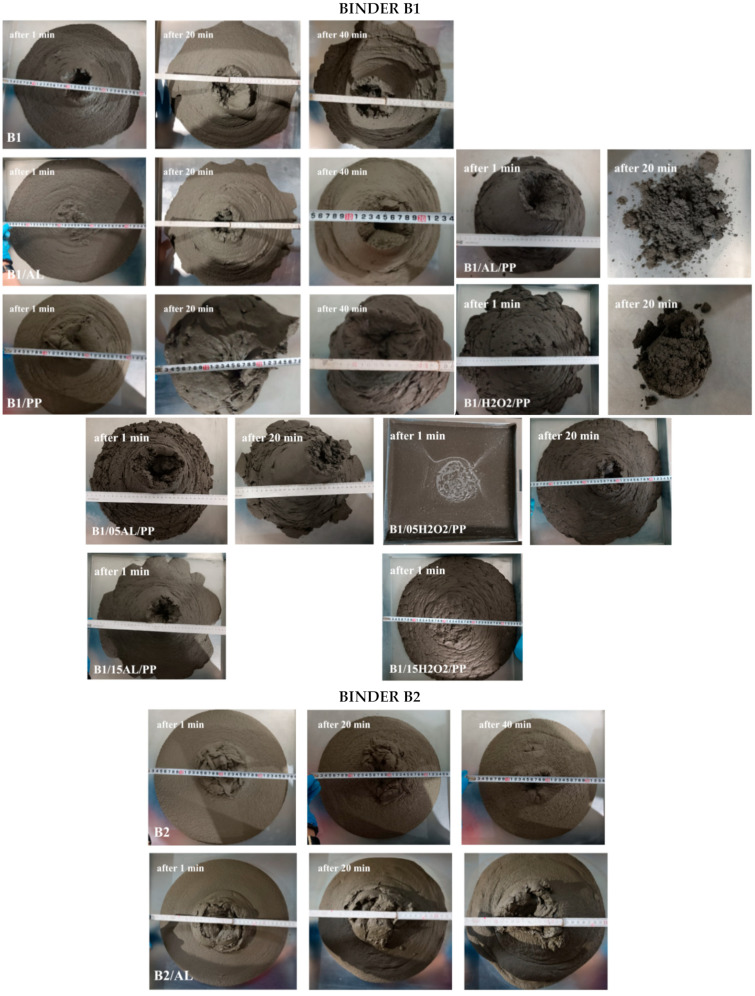

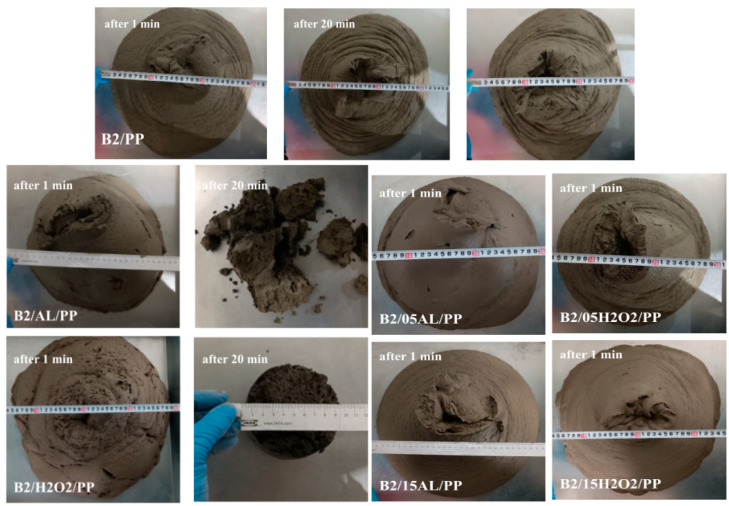
Testing the consistency of the mixtures and their stability and buildability.

**Figure 5:**
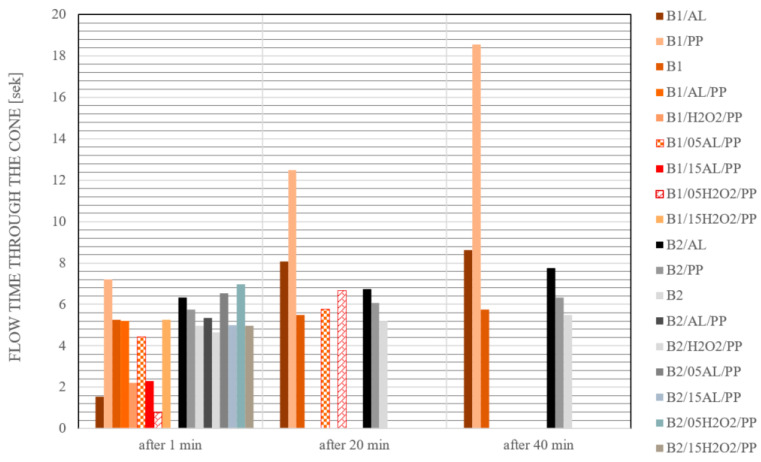
Flow time of the tested mixtures through the Abrams cone.

Figure 4 and Figure 5 present a representative picture of the consistency changes in the tested materials depending on their composition and mixing time. By comparing the recorded photographic evidence, a notable distinction was observed between the mixtures prepared with raw materials B1 and B2. Mixtures formulated with material B2 demonstrated superior stability and a homogeneous consistency throughout the entire volume. Each measurement revealed a uniformly compact structure with consistent and well-defined flow edges. Moreover, the layers formed during the removal of the Abrams cone when the material with the addition of polypropylene fibers was tested were repeatable. Conversely, the material prepared based on B1 behaved differently each time. Concrete without additives had an uneven flow edge and water precipitation. The addition of aluminum powder initially only slightly changed the consistency of the material, but after 40 min of mixing the material noticeably foamed. By contrast, the addition of hydrogen peroxide to the tested materials caused such a quick reaction with the concrete mixture (without fibers) that it was not possible to conduct a consistency test. Moreover, with the extension of the mixing time and the introduction of additives in the form of polypropylene fibers, the consistency of the mixtures became more and more compact (Table 5, Figure 4 and Figure 5). This effect was independent of the type of base raw material used.

It was observed that the B1/AL/PP and B1/H2O2/PP mixtures initially exhibited appropriate consistency post-mixing. However, conducting consistency tests after 20 min proved impractical due to the mixtures becoming excessively brittle, a condition deemed unacceptable. Furthermore, formulations containing 0.5% H_2_O_2_ exhibited excessive fluidity within the first minute of mixing, rendering them unsuitable for 3D concrete printing. After the first minute, B1/05H2O2/PP was excessively fluid, flowing through the cone in just 0.8 s. However, after 20 min of mixing, the flow time increased to 6.68 s. This rapid transformation in consistency suggests that the material undergoes swift alterations in its properties, making it unsuitable for additive manufacturing applications. Furthermore, the cone walls were heavily soiled by the mixture after the 20-min flow test. This indicates poor flow stability and residue formation, which would likely hinder the printing process and compromise the quality of the printed elements. The B1/05H2O2/PP mixture exhibited significantly different behavior compared to B1/05AL/PP. Conversely, the mixture incorporating 0.5% Al showed promising consistency both immediately after mixing and after the 20 min mark. The foamed concrete mixture B1/05AL/PP, reinforced with fibers, has demonstrated the most promising properties among the tested formulations. The flow time of this mixture through a cone was recorded at 4.43 s after 1 min and 5.78 s after 20 min, as illustrated in Figure 5. Although there is a noticeable increase, it is not significantly large, suggesting the mixture’s stability over time. The consistency of the mixture after 20 min is markedly different from that after 1 min, likely due to hydration processes and structural changes within the concrete (Figure 4). Moreover, after the mixture flows through the cone, no larger material elements, such as clumped fibers or layers of concrete, remain on the cone walls. This observation indicates that the material can seamlessly pass through the transportation system in the printer and through the nozzle during the printing process. In summary, the B1/05AL/PP mixture exhibits advantageous characteristics for applications in concrete printing. Its stable flow, marked by a minimal increase in flow time, and the absence of residues on the cone walls, highlight its potential to achieve a uniform structure in printed elements. These properties may enhance the efficiency and quality of concrete printing, making the B1/05AL/PP mixture a promising material for modern construction applications involving 3D printing technology. The realm of 3D concrete printing necessitates materials with extended workability periods. Considering the potential future applications of these materials, the findings are encouraging. Nevertheless, further investigations are warranted into the optimal proportions of the frother and fiber, as well as the printing window of the mixture. The tested materials were subjected to strength tests. The results of compressive and flexural strength depending on the type of tested materials were presented in Table 6 and Table 7, respectively.

Analysis of the compressive strength results of the samples made of the B1 and B2 mixtures showed differences in values depending on their composition (Table 6).

The reference samples of the B1 material containing silica fly dust exhibited an average compressive strength of 21.55 MPa. The addition of polypropylene fibers (B1/PP) resulted in significantly enhanced strength properties, with values peaking at 33.12 MPa. Furthermore, it was found that the choice of foaming agent distinctly influenced the compressive strength of the samples. Samples with the addition of aluminum powder as the foaming agent (B1/AL, B1/05AL/PP, B1/15AL/PP) demonstrated compressive strengths of 28.76 MPa, 10.77 MPa, and 3.43 MPa, respectively.

Conversely, the use of H_2_O_2_ instead of Al powder as a foaming agent significantly reduced the compressive strength of materials with similar compositions. For the B1/05H2O2/PP sample, almost half the compressive strength value was measured compared to its counterpart foamed with Al powder. Moreover, the B1/H2O2 sample had even greater differences, with almost three times lower properties, compared to the B1/Al sample. It is worth noting that the B1/AL/PP and B1/H2O2/PP mixtures exhibited the lowest compressive strengths among the tested formulations based on B1, with values of 1.99 MPa and 1.02 MPa, respectively. These results were consistent with the observed brittleness of the materials 20 min after preparation. A similar trend was noted in the bending strength tests (Table 7).

The compressive strength test results of the B2 samples showed significantly lower values compared to samples made based on the B1 raw material. Any modification to the B2 composition resulted in a reduction in compressive strength. The introduction of polypropylene (PP) fibers had the least detrimental effect. The B2/PP mixture achieved 10.89 MPa. However, attempts to foam the material resulted in drastic reductions in strength properties. The compressive strength of the B2/AL and B2/AL/PP mixtures were similar, registering 1.19 MPa and 1.35 MPa, respectively. A notable difference was observed based on the type of foaming agent used. Samples foamed with hydrogen peroxide (H_2_O_2_) displayed better strength values compared to those foamed with aluminum powder. This effect was opposite to the previously described properties of materials based on raw material B1. Despite this, the compressive strengths were still insufficient for practical applications. Such low values restrict the potential use of these materials in additive manufacturing, as they would not provide the necessary compressive strength required for successive layers in 3D printing.

Table 7 presents the flexural strength of samples based on the B1 and B2 materials depending on the composition and the foaming agent used. Based on the recorded results, the three-point bending strength of the B1 material without additives reached 4.72 MPa. Lower values were observed for samples foamed with hydrogen peroxide (B1/H2O2), with an average strength of 3.34 MPa. For both foamed and fiber-reinforced samples, a significant trend was observed, showing lower values compared to the reference mixtures. Specifically, the B1/AL/PP and B1/H2O2/PP mixtures demonstrated significantly lower bending strengths than samples where only the amount of frother or water was modified. The three-point bending strength of B2 concrete samples was lower compared to the B1 material, with an average value of 2.93 MPa. The highest bending strength for B2-based mixtures was recorded for the mixture with added polypropylene fibers, reaching 3.92 MPa. Foamed samples, regardless of the additive type, exhibited low bending strength values, averaging around 1 MPa. Surprisingly low values were recorded for samples with added fibers (B1/PP).

**Figure 6:**
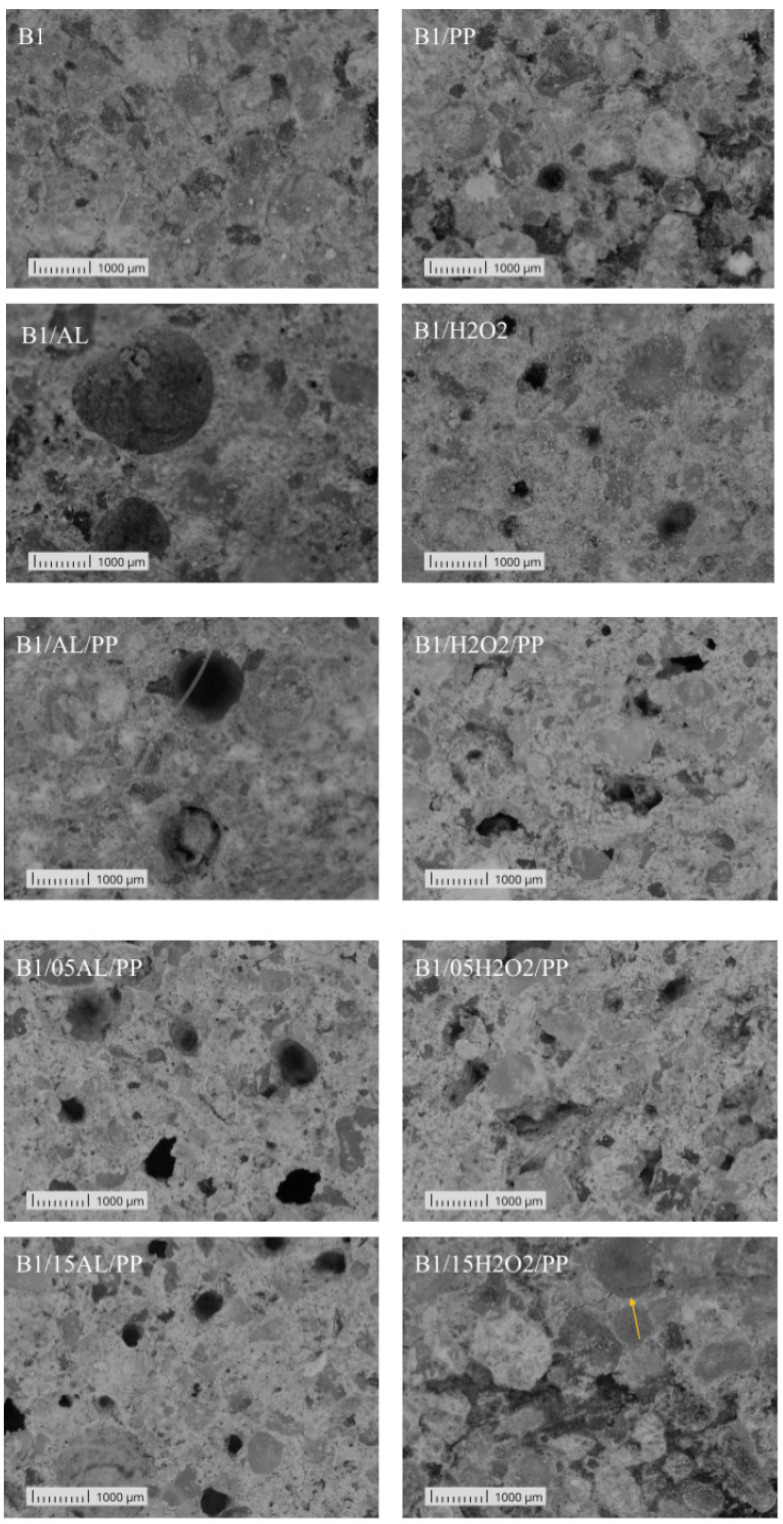
Representative microstructures of materials based on raw material B1: B1/AL, B1/H2O2, B1, B1/PP, B1/AL/PP, and B1/H2O2/PP. The arrow indicates a structural crack.

**Figure 7:**
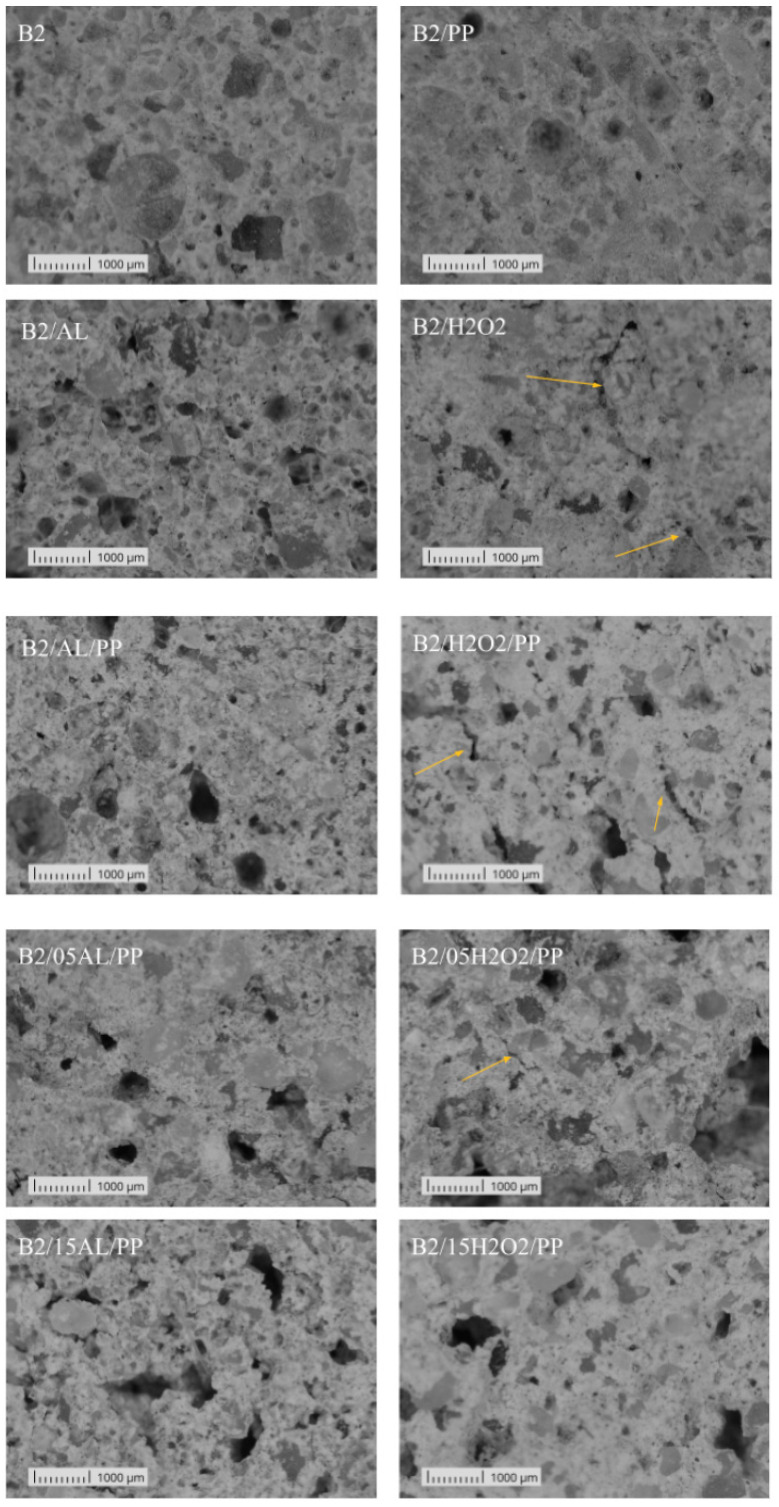
Representative microstructures of materials based on raw material B2: B2/AL, B2/H2O2, B2,B2/PP, B2/AL/PP, and B2/H2O2/PP. The arrows indicate structural cracks.

The microscopic observation revealed significant differences in the structure, size, and edges of the pores between the B1 and B2 materials (Figure 6 and Figure 7). In both cases, the pores created by foaming with aluminum powder were round. The B2 samples dis-played small, regular pores almost uniformly distributed throughout the entire volume, contrasting with the B1 samples, which had larger and less evenly distributed pores, indicating a lower degree of foaming. The sample B1/AL exhibits the largest pores, with the highest average pore surface area among the tested group. The regularity of the pore shape enhances the uniformity of the mechanical properties, potentially benefiting the material’s low thermal conductivity and overall mechanical performance. This pore configuration suggests a well-regulated production process. Similarly, samples B1/05AL/PP and B1/15AL/PP are characterized by a large number of regularly shaped pores. In the analysis of samples foamed with aluminum powder, it is noteworthy that only the sample B2/15AL/PP displays uneven edges of air bubbles. Samples foamed with hydrogen peroxide exhibited lower strength values and were characterized by small, densely packed pores with very irregular edges. Moreover, the B2-based materials showed a rougher microscopic structure. Additionally, cracks were visible in the structure of the B2/H2O2, B2/H2O2/PP, and B2/05H2O2/PP samples (marked with arrows). The B1/05H2O2/PP sample is characterized by the smallest average pore size and a high pore density. This phenomenon is likely associated with the relatively high density of the sample, which may be attributed to the fibers used during the production process, potentially leading to the penetration of air pores. A similar crack was observed in the B1-based material in the sample marked as B1/15H2O2/PP.

The density of samples made of the B2 material was characterized by significantly lower values compared to samples made of B1 raw material (Table 8). This difference highlights the importance of optimization and provides the ability to control the manufacturing process to tailor material properties to specific applications.

**Figure 8:**
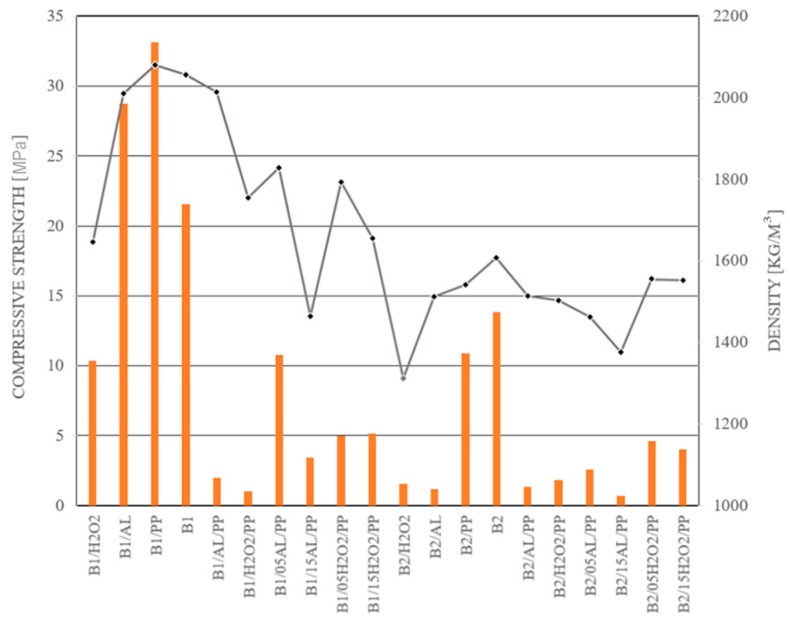
Characteristics of the tested materials in the context of the analysis of compressive strength and their density.

**Figure 9:**
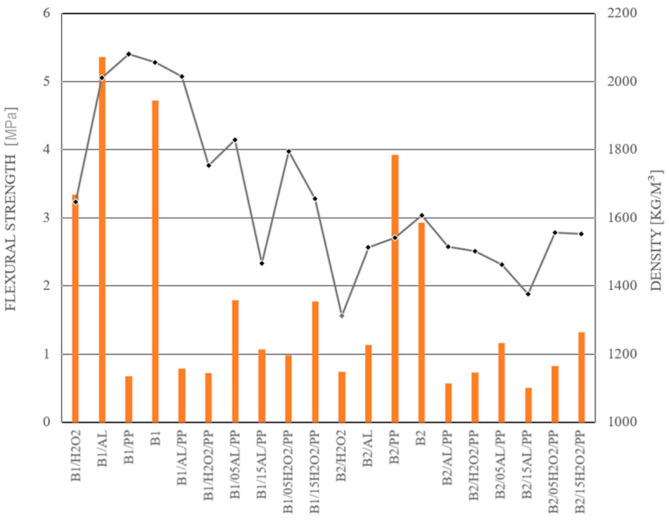
Characteristics of the tested materials in the context of the analysis of bending strength and their density.

The analysis of the literature indicates that the addition of foaming agents, such as aluminum powder and H_2_O_2_, significantly impacts cohesion and workability. The results obtained from observing the mixture flow through the Abrams cone and the concrete flow during testing reveal that, with minor exceptions (B1/05H_2_O_2_/PP and B1/15H_2_O_2_/PP), concrete foams initially exhibit lower consistency. Subsequently, their structure becomes significantly denser, and they demonstrate slower material spreading during tests, corroborating the findings of other researchers [51]. Furthermore, the comparative analysis of the mixtures in this study confirms that the addition of fibers in the tested materials increases water absorption [52]. The strength-to-density ratio of the tested samples is not always straightforward; higher material density does not consistently correlate with increased compressive or bending strength. An exemplary case is sample B1/H_2_O_2_, which, despite its low density, demonstrates the highest compressive and bending strength. Conversely, B1/H_2_O_2_/P and B1/AL/PP samples, which exhibit the highest density, do not achieve the highest strength values. The influence of additives on strength properties is substantial. Figure 8 and Figure 9 illustrate the correlation between the mixture’s density and its corresponding bending and compressive strengths.

The results of this study provide valuable insights into the structure and behavior of foamed concrete influenced by foaming agents, such as aluminum powder and hydrogen peroxide. The literature extensively describes the properties and outcomes achieved during experiments conducted by other researchers. However, there is a scarcity of studies detailing the behavior of the mixture itself during testing. The completed research demonstrates that, regardless of the amount of foaming chemicals added, a concrete mixture containing fibers is not feasible for use in 3D concrete printing. This finding aligns with numerous studies discussing 3D printing with foamed concrete using protein-based or synthetic foaming agents. The conducted studies demonstrate that 3D printing with foamed fiber-reinforced concrete is possible, but it requires a lot of research focused mainly on the behavior of the material in the context of 3D printing. Previous researchers have conducted experiments leading to the use of the material in additive technology by foaming concrete only with the use of foaming agents. This finding corroborates numerous studies discussing 3D printing with foamed concrete using protein-based or synthetic foaming agents. However, 3D printing with fiber-reinforced foamed concrete is theoretically possible, but it necessitates extensive research focused primarily on the behavior of the material specifically designed for 3D printing. A comprehensive understanding and optimization of the mixture’s properties are essential to ensure adequate consistency, homogeneity, and strength during the printing process [4,6,33,49,50,51,53,54,55].

## 4. Conclusions

In the consistency evaluation, by the slump flow method, compositions based on the B1 raw material demonstrated higher fluidity than those based on the B2 raw material. Modifying the composition of the mixtures by introducing fibers, the foaming process, and extending the mixing time influenced the change in consistency from liquid to dense plastic. This effect was independent of the type of raw material used. The content of foaming chemicals directly influences the bubble content in fresh foam mortar, as well as the pore structure of the hardened mortar, which is crucial for the rheological and hardening properties of 3D printed fiber-reinforced concrete. Mixtures with double the foaming agent content, i.e., 0.075% (B1/05AL/PP, B1/05H2O2/PP, B2/05AL/PP, B2/05H2O2/PP), demonstrated higher compressive and bending strengths with lower densities compared to the initial combined mixtures (B1/AL/PP, B1/H2O2/PP, B2/AL/PP, and B2/H2O2/PP). To utilize these materials effectively in 3D printing, additives should be incorporated to stabilize the mixture and extend its open time, ensuring better printability. Mixtures containing twice the amount of network water exhibited significantly altered rheological properties compared to fresh foam mortar. Despite their increased strength values, these mixtures proved to be very unstable for further testing in additive manufacturing technology. Moreover, B1/AL/PP and B1/H2O2/PP mixtures became too brittle after 20 min of mixing, rendering them unsuitable for 3D concrete printing technology. The B1/05H2O2/PP mixture initially exhibited excessive fluidity, flowing through the cone in just 0.8 s after the first minute. However, its flow time increased significantly to 6.68 s after 20 min of mixing. After the 20 min flow test, the cone walls were heavily soiled by the B1/05H2O2/PP mixture, indicating poor flow stability and residue formation. These characteristics suggest that the material has a narrow optimal application window, likely hindering the printing process and compromising the quality of the printed elements. Generally, the strength properties of samples from raw material B1 were significantly higher than those made from B2. Moreover, the foaming process further reduces the strength properties of the samples. The optimal results were achieved with the B1/H₂O₂ and B1/0.5AL/PP compositions. Microscopic analysis revealed significant differences in the pore structures of B1 and B2 materials. Samples foamed with aluminum powder had round, regular-shaped pores, while those foamed with hydrogen peroxide had small, densely packed, irregular pores. The irregular and densely packed pores in samples formed with hydrogen peroxide are consistent with previous findings by other researchers, who reported that hydrogen peroxide can produce a more irregular pore structure. The lower strength values observed in these samples align with the literature indicating that irregular pores can compromise the material’s mechanical integrity [56,57]. Further continuation of research on the development of more ecological concrete mixtures, e.g., from the B1 raw material, is crucial for the possibility of their use in additive technologies. Specifically, the open time for printing must exceed 20 min, and the material must achieve the desired porosity while maintaining high compressive and bending strength. This optimization is essential for validating the material’s performance and suitability for 3D printing applications.

## Figures and Tables

**Table 1:** Physical properties of the aluminum powder [35].

Active Aluminum Content [%]	Water Coverage [cm^2^/g]	Particle Size Distribution [μm]	Bulk Density [g/cm^3^]
≥93	ca. 7300	102–119	0.16

**Table 2:** Physical properties of the hydrogen peroxide H_2_O_2_ [36].

Solubility in Water	Boiling Point	Melting Point	Density [g/cm^3^]
Miscible	150 °C	–0.43 °C	1.13

**Table 3:** Physical properties of the polypropylene fibers [37].

Water Absorption (%)	Tensile Strength [N/mm^2^]	Young’s Modulus [N/mm^2^]	Density [g/cm^3^]
0	420	3800	0.91

**Table 4:** Designation of the investigated samples depending on the composition and the foaming agent used.

No.	Designation of the Mixtures	Cementitious Binder [%]		PP-12 [%]	Foaming Admixture [%]		Water [%]
1	B1/H2O2	B1	84.53	-	H_2_O_2_	2.86	12.61
2	B1/AL		87.00	-	Al	0.15	12.85
3	B1/PP		86.62	0.40	-	-	12.98
4	B1		87.23	-	-	-	12.77
5	B1/AL/PP		86.60	0.40	Al	0.15	12.85
6	B1/H2O2/PP		84.10	0.40	H_2_O_2_	3.30	12.20
7	B1/05AL/PP		86.68	0.40	Al	0.075	12.85
8	B1/15AL/PP		80.85	0.40	Al	0.15	18.60
9	B1/05H2O2/PP		85.90	0.40	H_2_O_2_	1.50	12.20
10	B1/15H2O2/PP		80.03	0.40	H_2_O_2_	3.30	16.27
11	B2/H2O2	B2	80.09	-	H_2_O_2_	3.51	16.40
12	B2/AL		82.37	-	Al	0.15	17.48
13	B2/PP		82.12	0.40	-	-	17.48
14	B2		83.10	-	-	-	16.90
15	B2/AL/PP		82.15	0.40	Al	0.15	17.60
16	B2/H2O2/PP		79.70	0.40	H_2_O_2_	3.10	16.80
17	B2/05AL/PP		81.93	0.40	Al	0.075	17.60
18	B2/15AL/PP		79.29	0.40	Al	0.15	20.16
19	B2/05H2O2/PP		81.85	0.40	H_2_O_2_	0.15	17.60
20	B2/15H2O2/PP		79.50	0.40	H_2_O_2_	3.10	17.00

**Table 5:** Slump flow test results depending on the composition and the foaming agent used.

No.	Designation of the Mixtures	Slump Flow Test		
		1 min [mm]	20 min [mm]	40 min [mm]
1	B1/H2O2	nd *	nd *	nd *
2	B1/AL	455	432	420
3	B1/PP	340	315	300
4	B1	458	435	420
5	B1/AL/PP	340	nd *	nd *
6	B1/H2O2/PP	465	nd *	nd *
7	B1/05AL/PP	445	370	nd *
8	B1/15AL/PP	415	nd *	nd *
9	B1/05H2O2/PP	full	460	nd *
10	B1/15H2O2/PP	480	nd *	nd *
11	B2/H2O2	nd *	nd*	nd *
12	B2/AL	400	320	290
13	B2/PP	370	345	320
14	B2	400	375	375
15	B2/AL/PP	325	nd *	nd *
16	B2/H2O2/PP	440	nd *	nd *
17	B2/05AL/PP	390	nd *	nd *
18	B2/15AL/PP	385	nd *	nd *
19	B2/05H2O2/PP	330	nd *	nd *
20	B2/15H2O2/PP	430	nd *	nd *

* The result cannot be presented due to the lack of fluidity of the mixture.

**Table 6:** Compressive strength of samples based on the B1 and B2 materials depending on the composition and the foaming agent used.

No.	Designation of the Mixtures	Compressive Strength [MPa]	Standard Deviation [MPa]
1	B1/H2O2	10.37	0.49
2	B1/AL	28.76	2.17
3	B1/PP	33.12	1.47
4	B1	21.55	2.68
5	B1/AL/PP	1.99	0.80
6	B1/H2O2/PP	1.02	0.22
7	B1/05AL/PP	10.77	0.56
8	B1/15AL/PP	3.43	0.67
9	B1/05H2O2/PP	4.98	0.32
10	B1/15H2O2/PP	5.14	0.36
11	B2/H2O2	1.58	0.18
12	B2/AL	1.19	0.24
13	B2/PP	10.89	1.46
14	B2	13.87	0.25
15	B2/AL/PP	1.35	0.12
16	B2/H2O2/PP	1.85	0.21
17	B2/05AL/PP	2.59	0.21
18	B2/15AL/PP	0.72	0.09
19	B2/05H2O2/PP	4.62	0.29
20	B2/15H2O2/PP	4.04	0.26

**Table 7:** Flexural strength of samples based on the B1 and B2 materials depending on the composition and the foaming agent used.

No.	Designation of the Mixtures	Flexural Strength [MPa]	Standard Deviation [MPa]
1	B1/H2O2	3.34	0.18
2	B1/AL	5.36	0.96
3	B1/PP	0.67	0.15
4	B1	4.72	0.49
5	B1/AL/PP	0.79	0.05
6	B1/H2O2/PP	0.72	0.11
7	B1/05AL/PP	1.79	0.45
8	B1/15AL/PP	1.07	0.12
9	B1/05H2O2/PP	0.98	0.14
10	B1/15H2O2/PP	1.77	0.12
11	B2/H2O2	0.74	0.18
12	B2/AL	1.13	0.15
13	B2/PP	3.92	0.23
14	B2	2.93	0.47
15	B2/AL/PP	0.57	0.18
16	B2/H2O2/PP	0.73	0.23
17	B2/05AL/PP	1.16	0.16
18	B2/15AL/PP	0.50	0.11
19	B2/05H2O2/PP	0.82	0.18
20	B2/15H2O2/PP	1.32	0.24

**Table 8:** Density of samples based on the B1 and B2 materials depending on the composition and the foaming agent used.

No.	Designation of the Mixtures	Density [kg/m^3^]	Standard Deviation [kg/m^3^]
1	B1/H2O2	1646.7	0.59
2	B1/AL	2009.9	0.54
3	B1/PP	2079.9	0.41
4	B1	2055.7	0.40
5	B1/AL/PP	2014.0	0.60
6	B1/H2O2/PP	1754.1	0.59
7	B1/05AL/PP	1828.6	0.40
8	B1/15AL/PP	1465.3	0.52
9	B1/05H2O2/PP	1794.1	0.46
10	B1/15H2O2/PP	1656.2	0.47
11	B2/H2O2	1312.1	0.60
12	B2/AL	1512.5	0.62
13	B2/PP	1541.9	0.55
14	B2	1607.5	0.39
15	B2/AL/PP	1514.6	0.54
16	B2/H2O2/PP	1502.3	0.52
17	B2/05AL/PP	1462.9	0.41
18	B2/15AL/PP	1375.7	0.42
19	B2/05H2O2/PP	1555.5	0.49
20	B2/15H2O2/PP	1552.2	0.59

## Data Availability

No new data were created or analyzed in this study. Data sharing is not applicable to this article.
